# Macrophage Differentiation from Monocytes Is Influenced by the Lipid Oxidation Degree of Low Density Lipoprotein

**DOI:** 10.1155/2015/235797

**Published:** 2015-07-29

**Authors:** Jin-Won Seo, Eun-Jeong Yang, Kyung-Hwa Yoo, In-Hong Choi

**Affiliations:** ^1^Department of Microbiology, Institute for Immunology and Immunological Diseases, Brain Korea 21 PLUS Project for Medical Science, Yonsei University College of Medicine, Seoul 120-752, Republic of Korea; ^2^Department of Physics, Yonsei University, Seoul 120-752, Republic of Korea

## Abstract

LDL plays an important role in atherosclerotic plaque formation and macrophage differentiation. However, there is no report regarding the oxidation degree of LDL and macrophage differentiation. Our study has shown that the differentiation into M1 or M2 macrophages is related to the lipid oxidation level of LDL. Based on the level of lipid peroxidation, LDL is classified into high-oxidized LDL (hi-oxLDL) and low-oxidized LDL (low-oxLDL). The differentiation profiles of macrophages were determined by surface receptor expression and cytokine secretion profiles. Low-oxLDL induced CD86 expression and production of TNF-*α* and IL-12p40 in THP-1 cells, indicating an M1 macrophage phenotype. Hi-oxLDL induced mannose receptor expression and production of IL-6 and monocyte chemoattractant protein-1, which mostly match the phenotype of M2 macrophages. Further supporting evidence for an M2 polarization by hi-oxLDL was the induction of LOX-1 in THP-1 cells treated with hi-oxLDL but not with low-oxLDL. Similar results were obtained in primary human monocytes. Therefore, our results strongly suggest that the oxidation degree of LDL influences the differentiation of monocytes into M1 or M2 macrophages and determines the inflammatory fate in early stages of atherosclerosis.

## 1. Introduction

Oxidized low-density lipoprotein (oxLDL) plays a critical role in atherosclerotic plaque formation, which is triggered by the persistence of lipid-laden macrophages [1] as well as interfering endothelial cell motility [2] in arterial wall. The differentiation of monocytes into macrophages, which accumulate oxLDL to form foam cells, is induced by oxLDL [1, 3]. The process starts with endothelial activation in the subendothelial areas by oxLDL deposition [4, 5]. Monocytes migrate into the intima guided by chemokines [6] and differentiate into macrophages. These macrophages then take up modified lipoproteins and, as they accumulate excess lipids, they form foam cells [3]. The foam cells die and release intracellular contents [7], which induce an inflammatory reaction. This in turn attracts more macrophages into the lesion. During this process, LDL may be oxidized by enzymes, for example, myeloperoxidase [8, 9], or by reactive oxygen species (ROS) [3] in the inflammatory microenvironment of the plaque. Although oxLDL is important for atherosclerotic inflammation, there is no report about the oxidation degree of LDL and macrophage differentiation.

Macrophages are fundamental contributors in the development and progression of atherosclerosis. Monocytes that are recruited into the intima are exposed to a variety of stimuli, such as cytokines, lipids, iron, and calcium, which exist in complex microenvironments. These factors can influence the phenotypic polarization of macrophages and their subsequent activation [10]. Classically activated (M1) macrophages and alternatively activated (M2) macrophages are well-defined phenotypes. In addition, recent studies suggested that several subtypes of M2 macrophages (M2a, b, c, and d) and stimuli-induced subpopulations such as Mox, Mhem, M (Hb), and M4 macrophages exist in atherosclerotic lesions [11].

Therefore, we hypothesized that LDL with different oxidation levels exists in the early atherosclerotic milieu and that the oxidation degree of LDL influences macrophage differentiation into classically activated (M1) or alternatively activated (M2) macrophages, which determines the inflammatory fate for atherosclerotic development. In this study, monocytes were treated with high-oxidized LDL (hi-oxLDL) or low-oxidized LDL (low-oxLDL) and their surface receptor expression and cytokine secretion profiles were analyzed to determine if they differentiated into M1 or M2 macrophages.

## 2. Materials and Methods

### 2.1. Reagents

Human native LDL and oxidized LDL were purchased from Biomedical Technologies Inc. (Stoughton, MA, USA). During electrophoresis, low-oxLDL migrates further than the native LDL and is also defined by a low TBAR value (8.7–11.8 nmol/mg protein). Hi-oxLDL migrates 2.7 fold further than the native LDL and is defined by a high TBAR value (55.7–75.6 nmol/mg protein). Anti-human CD86-PE and anti-human CD206- (mannose receptor-) FITC antibodies were purchased from Beckman Coulter Inc. (Brea, CA, USA) and BD Biosciences (San Jose, CA, USA), respectively. Anti-LOX-1 and anti-tubulin antibodies were purchased from Abcam (Cambridge, MA, USA) and BioLegend (San Diego, CA, USA), respectively. Recombinant human M-CSF was purchased from BioLegend (San Diego, CA, USA). LPS (*E. coli* O26:B6) was purchased from Sigma-Aldrich (St. Louis, MO, USA).

### 2.2. Culture of THP-1 Cells

The human monocytic THP-1 cell line was purchased from ATCC and cultured in RPMI1640 medium containing 10% FBS and penicillin-streptomycin (100 units/mL and 100 *μ*g/mL, resp.) at 37°C in a humidified 5% CO_2_ incubator.

### 2.3. Analysis of Cell Proliferation

The cell proliferation rate was measured using the colorimetric cell counting kit-8 (CCK-8) (Dojindo laboratories, Kyoto, Japan). THP-1 cells were cultured at 1 × 10^5^ cells per well in 24-well plates and treated with native LDL, low-oxLDL, or hi-oxLDL (50 *μ*g/mL each). After 24, 48, or 96 h, the CCK-8 reagent was added to each well and cells were incubated for 30 min at 37°C. The supernatant was harvested and transferred to 96-well plates. The O.D. at 450 nm was determined with a spectrophotometer.

### 2.4. Flow Cytometry

THP-1 cells were cultured at 2 × 10^5^ cells per well in 12-well plates and treated with native LDL, low-oxLDL, or hi-oxLDL (50 *μ*g/mL each). After 24 or 96 h, cells were harvested and analyzed using flow cytometry to measure granularity and autofluorescence. To detect surface markers, cells were treated with native LDL, low-oxLDL, or hi-oxLDL (50 *μ*g/mL each) for 96 h and stained with PE-conjugated anti-human CD86 antibodies. Flow cytometric analysis was performed by a BD LSR II flow cytometer (BD Bioscience).

### 2.5. Confocal Microscopy

THP-1 cells were cultured in 2-well chambered cover glasses at 2 × 10^5^ cells per well and treated with native LDL, low-oxLDL, or hi-oxLDL (50 *μ*g/mL each) for 96 h. Attached cells were stained with FITC-conjugated anti-human mannose receptor (CD206) antibodies and analyzed using an FV1000 microscope (Olympus, Tokyo, Japan).

### 2.6. Isolation of Human Monocytes and Observation of Morphological Changes

Blood was obtained from healthy donors after acquiring internal review board approval and informed consent (number 4-2012-0088). Peripheral blood mononuclear cells (PBMCs) were separated by Ficoll-Hypaque density gradient centrifugation (density = 1.077) at 1,600 rpm for 30 min and human monocytes were purified from PBMCs using Monocyte Isolation Kit II (Miltenyi Biotec, Bergsch Gladbach, Germany). Staining for CD14 was performed to confirmed monocyte population and usually >95% of cells were positive. Human monocytes were cultured in RPMI1640 medium containing 10% FBS and penicillin-streptomycin (100 units/mL and 100 *μ*g/mL, resp.) at 37°C in a humidified 5% CO_2_ incubator. Human monocytes were cultured in 12-well plates at 2 × 10^5^ cells per well and incubated with both M-CSF (50 ng/mL) and hi-oxLDL or low-oxLDL (50 *μ*g/mL each) for 7 days. Morphological changes were observed using bright-field microscope (IX71, Olympus, Tokyo, Japan).

### 2.7. Enzyme-Linked Immunosorbent Assay (ELISA)

THP-1 cells were cultured at 2 × 10^5^ cells per well in 12-well plates and pretreated with native LDL, low-oxLDL, or hi-oxLDL (50 *μ*g/mL each) for 24, 48, or 96 h. The medium was replaced with fresh medium and then cells were stimulated with LPS (100 ng/mL). Primary monocytes were cultured at 2 × 10^5^ cells per well in 12-well plates and pretreated with both M-CSF (50 ng/mL) and native LDL, low-oxLDL, or hi-oxLDL (50 *μ*g/mL each) for 24, 48, or 72 h. Primary monocytes were stimulated with LPS (20 ng/mL). Eighteen hours after LPS stimulation, culture supernatants were harvested and stored at −80°C. ELISA was performed with a human cytokine TNF-*α*, IL-12p40, IL-6, and monocyte chemoattractant protein-1 (MCP-1) assay kit (BD Bioscience). The O.D. at 450 nm was determined.

### 2.8. Western Blot

THP-1 cells were seeded at 2 × 10^5^ cells per well and treated with native LDL, low-oxLDL, or hi-oxLDL (50 *μ*g/mL each). After 24 and 48 h, cells were harvested and lysed with lysis buffer. After centrifugation, total protein was stored at −80°C. Each 50 *μ*g of protein samples was loaded in 12% SDS-PAGE and transferred onto nitrocellulose membrane (GE Healthcare, NJ, USA). Then, membranes were reacted with anti-LOX-1 or anti-tubulin antibodies. Protein bands were detected using a West Save Up western blot detection kit (Ab frontier, Seoul, Korea).

### 2.9. Statistical Analysis

One-way ANOVA analysis was conducted. *p* < 0.05 was considered significant.

## 3. Results

### 3.1. Differential Effects of the LDL Oxidation Degree on Granularity Induction and Proliferation of Monocytes

**Figure 1:**
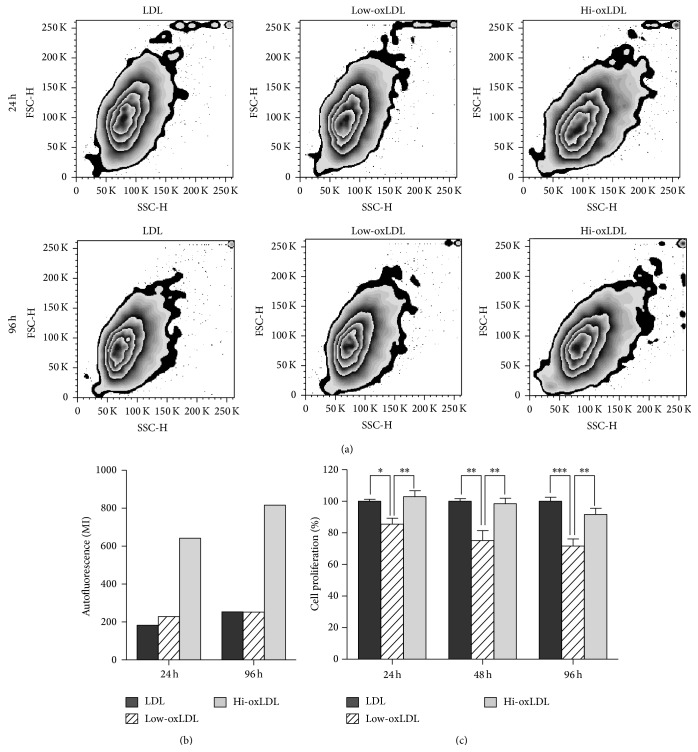
Effects of oxLDL on granularity, autofluorescence, and proliferation. THP-1 cells were treated with differentially oxidized LDL (50 *μ*g/mL) for 24, 48, or 96 h. (a) Granularity and (b) autofluorescence were measured by flow cytometer. (c) Cell proliferation was assessed by CCK-8 assay. Data represent means ± S.E. of three independent experiments. One-way ANOVA analysis was used to determine significance (^*∗*^ *p* < 0.05, ^*∗∗*^ *p* < 0.01, and ^*∗∗∗*^ *p* < 0.001).

To investigate the effects of the degree of LDL oxidation, we assessed differentiation and proliferation of THP-1 cells after treatment with native LDL, low-oxLDL, or hi-oxLDL. Cell granularity (Figure 1(a)) and autofluorescence (Figure 1(b)) were significantly increased in cells treated with hi-oxLDL compared to cells treated with native LDL or low-oxLDL. These effects by hi-oxLDL increased over time. After treatment with hi-oxLDL, the cell proliferation rate did not change compared to those of cells treated with native LDL. However, cell proliferation was significantly decreased in cells treated with low-oxLDL compared to that in cells treated with hi-oxLDL (Figure 1(c)). These results indicate that hi-oxLDL increases granularity and autofluorescence whereas low-oxLDL decreases cell proliferation, suggesting that monocytes respond differently to oxidation degree of LDL.

### 3.2. Effect of OxLDL on the Expression of Surface Markers of Macrophages

**Figure 2:**
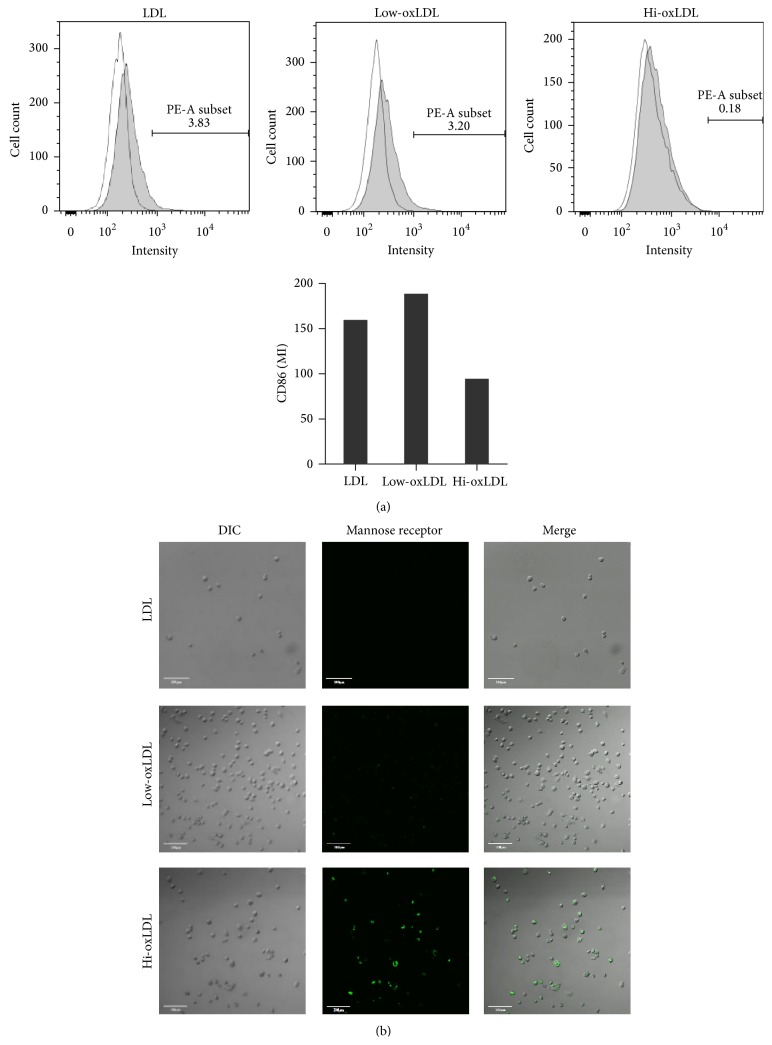
Expression of CD86 and CD206 in response to oxLDL. THP-1 cells were treated with oxLDL (50 *μ*g/mL) for 96 h. (a) THP-1 cells were stained with anti-CD86-PE antibodies and analyzed by flow cytometry. (b) THP-1 cells were stained with anti-CD206-FITC antibodies and observed by confocal microscope (×200).

Next, we tested whether the degree of LDL oxidation influences the differentiation of monocytes into macrophages. When cells were treated with low-oxLDL, the expression level of the marker for M1 macrophages, CD86, was increased compared to that in cells treated with hi-oxLDL (Figure 2(a)). In contrast, the expression level of the marker for M2 macrophages, mannose receptor (CD206), was significantly increased in cells treated with hi-oxLDL compared to that in cells treated with native LDL or low-oxLDL (Figure 2(b)). These results indicate that the degree of LDL oxidation affects the differentiation of monocytes into different subtypes of macrophages.

### 3.3. Effect of OxLDL on Cytokine Production

**Figure 3:**
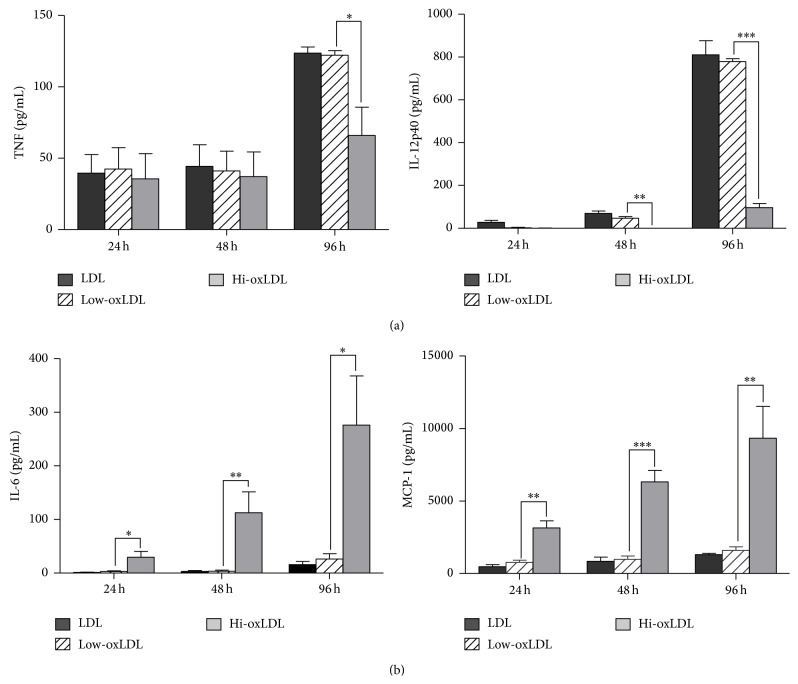
Production of cytokines and chemokines in response to oxLDL. THP-1 cells were pretreated with differentially oxidized LDL (50 *μ*g/mL) for 24, 48, or 96 h and treated with LPS (100 ng/mL) for 18 h. Culture supernatants were harvested and the levels of TNF-*α*, IL-12p40, IL-6, and MCP-1 were measured by ELISA. Data represent means ± S.E. of three independent experiments. One-way ANOVA analysis was used to determine significance (^*∗*^ *p* < 0.05, ^*∗∗*^ *p* < 0.01, and ^*∗∗∗*^ *p* < 0.001).

To assess cytokine production, THP-1 cells were preexposed to differentially oxidized LDL for 24, 48, or 96 h and then stimulated with LPS. The production of TNF-*α* and IL-12p40 was decreased in cells pretreated with hi-oxLDL compared to those in cells pretreated with low-oxLDL (Figure 3(a)). However, the production of IL-6 and MCP-1 was significantly increased in cells pretreated with hi-oxLDL compared to cells pretreated with low-oxLDL (Figure 3(b)). These results suggest that macrophages secrete different sets of cytokines depending on the oxidation level of LDL. More interestingly, low-oxLDL and hi-oxLDL induced opposite cytokine profiles.

### 3.4. Effects of OxLDL on LOX-1 Expression

**Figure 4:**
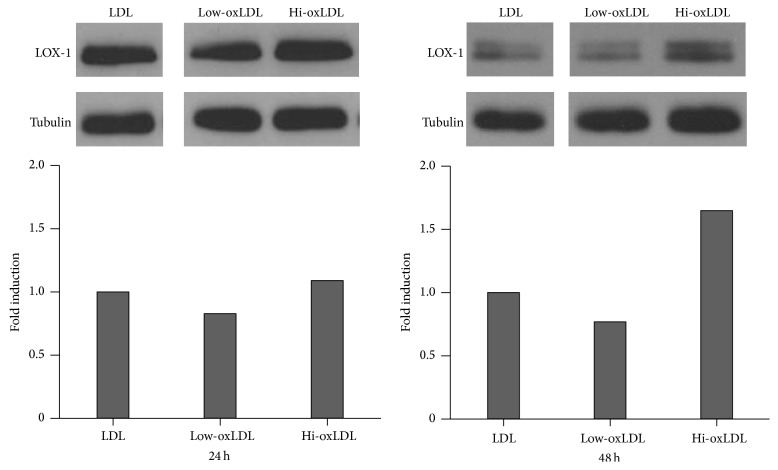
Expression of LOX-1 in response to oxLDL. THP-1 cells were treated with differentially oxidized LDL (50 *μ*g/mL) for 24 or 48 h. Each 50 *μ*g of protein samples was loaded and analyzed with anti-LOX-1 antibodies and anti-tubulin antibodies (internal control).

OxLDL binds to scavenger receptors, such as LOX-1, and also induces the expression of LOX-1 in macrophages. We tested the effect of oxLDL on the expression of LOX-1 in THP-1 cells. LOX-1 expression increased in cells treated with hi-oxLDL compared to cells treated with native LDL or low-oxLDL after 24 or 48 h (Figure 4). The expression of LOX-1 was slightly but not significantly decreased in cells treated with low-oxLDL compared to that in cells treated with native LDL (Figure 4).

### 3.5. Effects of OxLDL on Morphological Changes and Cytokine Production in Primary Monocytes

**Figure 5:**
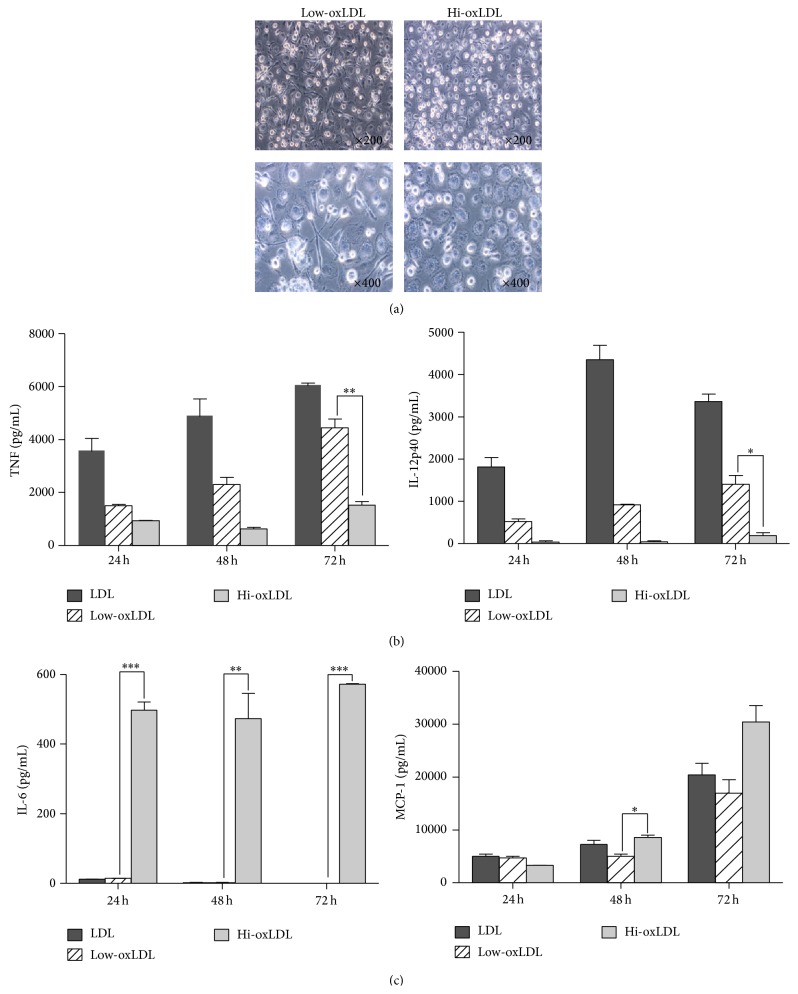
Morphological changes and cytokine production in primary monocytes. (a) Human blood monocytes were isolated from PBMCs and cultured with both M-CSF (50 ng/mL) and differentially oxidized LDL (50 *μ*g/mL each) for 7 days. Morphological changes were observed using bright-field microscope (×200, 400). (b) Primary monocytes were pretreated with both M-CSF (50 ng/mL) and differentially oxidized LDL (50 *μ*g/mL) for 24, 48, or 72 h and treated with LPS (20 ng/mL) for 18 h. Culture supernatants were harvested and the levels of TNF-*α*, IL-12p40, IL-6, and MCP-1 were measured by ELISA. Data represent means ± S.E. and one-way ANOVA analysis was used to determine significance (^*∗*^ *p* < 0.05, ^*∗∗*^ *p* < 0.01, and ^*∗∗∗*^ *p* < 0.001).

When human monocytes were treated with low-oxLDL, cells have changed into spindle-like shape, which is a unique feature of M1 macrophages whereas the treatment of hi-oxLDL led to round cells, which represents M2 macrophages (Figure 5(a)). The production of TNF-*α* and IL-12p40 was decreased in monocytes pretreated with hi-oxLDL compared to cells pretreated with low-oxLDL (Figure 5(b)). Moreover, the production of IL-6 and MCP-1 was significantly increased in monocytes pretreated with hi-oxLDL (Figure 5(c)). These results support that the oxidation degree of LDL affects primary monocytes in a similar way to the monocytic cell line, THP-1 cells.

## 4. Discussion

During atherosclerotic plaque development, LDL can be oxidized to various degrees by ROS or enzymes released by macrophages in the subendothelial area [3]. This modified LDL, along with growth factors such as M-CSF, affects differentiation of monocytes into macrophages. Depending on the extent and duration of oxidation, the physicochemical properties of LDL, such as the charge, particle size, and lipid content, are changed [12]. In addition, depending on whether the lipid or protein component is oxidized, oxidized LDL can induce different signaling pathways [13]. Our study investigated the effects of differentially oxidized LDL on the differentiation of macrophages. We used two types of oxLDL, which were classified as low-oxLDL or hi-oxLDL based on the TBAR value for lipid peroxidation and relative electrophoretic mobility of LDL particles on agarose gels [14, 15].

OxLDL is known to stimulate cell proliferation and induces differentiation of monocytes and macrophages [16, 17]. Treatment of THP-1 cells with hi-oxLDL led to an increase in cell granularity and autofluorescence, which is a feature of differentiated macrophages [18]. Moreover, hi-oxLDL did not change the cell proliferation rate compared to native LDL. In contrast, in cells treated with low-oxLDL, the granularity and autofluorescence were not increased and cell proliferation was significantly decreased (Figure 1). Next, as shown in Figures 2(a) and 2(b), CD86 (M1 macrophages) was induced by low-oxLDL whereas mannose receptor (M2 macrophages) was induced by hi-oxLDL. The results of the cytokine profiling showed an increased production of TNF-*α* as well as IL-12p40 (Figure 3(a)), which reflects the typical phenotype of M1 macrophages [19], whereas increased production of IL-6 and MCP-1 (Figure 3(b)) reflects the M2 macrophage phenotype. M2 macrophages can be further classified into four subtypes, namely, M2a, M2b, M2c, and M2d. Among them, M2b macrophages secrete high levels of IL-6 and are referred to as “regulatory macrophages” [10]. IL-6 is a pleiotropic cytokine that induces either proinflammatory or antiinflammatory responses and is involved in a variety of conditions, such as obesity, arthritis, colitis, and sepsis. Generally, IL-6 is considered a proinflammatory cytokine, but its antiinflammatory roles have been suggested in specific conditions [20]. Recent studies have shown that IL-6 promotes M2 activation while attenuating M1 activation. When myeloid cells were primed with IL-6, the STAT3-IL-4-STAT6 axis, which is a potent inducer of M2 polarization, was activated, whereas when monocytes were pretreated with IL-6, lipopolysaccharide-induced production of proinflammatory cytokines was attenuated [21]. Moreover, IL-6 induces the polarization of tumor-associated macrophages, which are similar to obesity-associated M2 macrophages [22]. These observations indicate that IL-6 plays a “counter-inflammatory” role in controlling metabolic homeostasis in low grade and chronic conditions, such as obesity.

MCP-1 plays an important role in the recruitment of circulating monocytes to inflamed sites. MCP-1-mediated recruitment of monocytes contributes to pathogenesis, but several studies have shown that MCP-1 has a beneficial role in various inflammatory conditions. In the gut, lamina propria macrophages, which revealed an M2-like phenotype, produce large amounts of MCP-1. Additionally, a subset of lamina propria macrophages are recruited in response to MCP-1 and produce a large amount of IL-10 to maintain gut homeostasis in the steady state as well as terminate excess inflammation in the intestine [23]. Furthermore, MCP-1 exerts beneficial effects in other inflammatory diseases such as ischemic heart diseases by inhibiting the generation of ROS and by healing necrotic areas in the myocardium [24, 25].

The exact roles of IL-6 and MCP-1 are still controversial. However, previous studies on the anti-inflammatory effects of IL-6 and MCP-1 support our results that hi-oxLDL-induced differentiation of macrophages skews towards the M2-like phenotype rather than the M1 phenotype (Figures 2(b) and 3(b)).

Our results show that hi-oxLDL induces IL-6 and MCP-1 production in macrophages, indicating that the expression of these cytokines may be a feature of M2 macrophages. While the interpretation of cytokine profiles might be difficult, most of our results for cytokine production and surface marker expression suggest that low-oxLDL induces M1 polarization, whereas hi-oxLDL leads to M2 polarization.

Next, we assessed the expression of LOX-1. LOX-1 is a receptor for oxLDL and is expressed in a variety of cells, such as endothelial cells, muscle cells, monocytes, and macrophages [2, 26, 27]. After binding to oxLDL, LOX-1 is internalized; this induces endoplasmic reticulum (ER) stress that causes upregulation of LOX-1, which occurs via a positive feedback loop between ER stress and LOX-1 expression and macrophage differentiation into M2 macrophages together with foam cell formation [27–29]. Figure 4 shows that treatment with hi-oxLDL increased the LOX-1 expression of macrophages. These high LOX-1 levels amplify the signals from hi-oxLDL and thus induce increased differentiation into M2 macrophages. A report using mesenchymal stem cells has demonstrated that treatments with oxLDL markedly increase expressions of LOX-1 and MCP-1 [30], but in which the oxidation degree of LDL was not clearly defined.

Lastly, we investigated the effects of differentially oxidized LDL in primary monocytes. Our study showed primary monocytes have changed into spindle shape after treatment with low-oxLDL and into round cells after treatment with hi-oxLDL. It is well known that the shape of M1 macrophages adopt more spindle-like shape, whereas M2 macrophages are more rounded [31, 32]. In addition to the morphological changes, the cytokines profiles and the decreased production of TNF-*α* and IL-12p40 as well as increased production of IL-6 and MCP-1 after treatment with hi-oxLDL (Figure 5(b)) strongly support the role of differentially oxidized LDL in monocyte differentiation to M1 or M2 macrophages.

**Figure 6:**
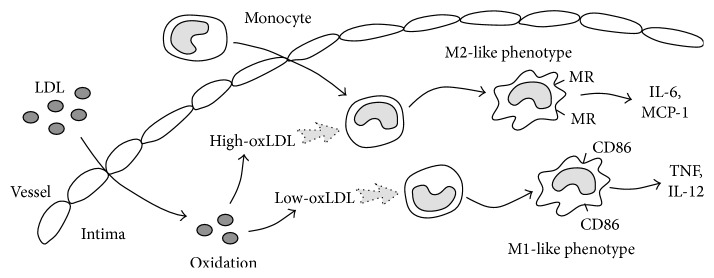
Graphical summary of monocyte differentiation into M1 or M2 macrophages by differentially oxidized LDL.

Taken together, our results for granularity, autofluorescence, cytokine profiles, and surface makers suggest that low-oxLDL induces macrophage polarization towards M1 phenotype whereas hi-oxLDL induces M2 phenotype. As summarized in Figure 6, our results suggest that the degree of oxidation of LDL is the important factor for differentiation of monocytes and macrophages and may determine the inflammation status or even the development of atherosclerosis at early stages of the disease.
